# Chronic traumatic encephalopathy (CTE): criteria for neuropathological diagnosis and relationship to repetitive head impacts

**DOI:** 10.1007/s00401-023-02540-w

**Published:** 2023-02-10

**Authors:** Ann C. McKee, Thor D. Stein, Bertrand R. Huber, John F. Crary, Kevin Bieniek, Dennis Dickson, Victor E. Alvarez, Jonathan D. Cherry, Kurt Farrell, Morgane Butler, Madeline Uretsky, Bobak Abdolmohammadi, Michael L. Alosco, Yorghos Tripodis, Jesse Mez, Daniel H. Daneshvar

**Affiliations:** 1 VA Boston Healthcare System, U.S. Department of Veteran Affairs, Boston, MA USA; 2 Boston University Alzheimer’s Disease Research Center and CTE Centers, Department of Neurology, Boston University School of Medicine, 150 S Huntington Ave, Boston, MA 02130 USA; 3 Department of Neurology, Boston University School of Medicine, Boston, MA USA; 4 Department of Pathology and Laboratory Medicine, Boston University School of Medicine, Boston, MA USA; 5 VA Bedford Healthcare System, Bedford, MA USA; 6 Department of Biostatistics, Boston University School of Public Health, Boston, MA USA; 7 Department of Epidemiology, Boston University School of Public Health, Boston, MA USA; 8 Departments of Pathology, Neuroscience, and Artificial Intelligence and Human Health, Neuropathology Brain Bank and Research Core, Ronald M. Loeb Center for Alzheimer’s Disease, Friedman Brain Institute, Icahn School of Medicine at Mount Sinai, New York, NY USA; 9 Department of Pathology and Laboratory Medicine, University of Texas Health Science Center at San Antonio, San Antonio, TX USA; 10 Glenn Biggs Institute for Alzheimer’s and Neurodegenerative Diseases, University of Texas Health Science Center at San Antonio, San Antonio, TX USA; 11 Department of Neuroscience, Mayo Clinic, Jacksonville, FL USA; 12 Department of Physical Medicine and Rehabilitation, Harvard Medical School, Boston, MA USA; 13 Department of Physical Medicine and Rehabilitation, Massachusetts General Hospital, Boston, MA USA; 14 Department of Physical Medicine and Rehabilitation, Spaulding Rehabilitation Hospital, Boston, MA USA

**Keywords:** CTE, Tauopathy, Repetitive head impacts, Neurodegeneration

## Abstract

Over the last 17 years, there has been a remarkable increase in scientific research concerning chronic traumatic encephalopathy (CTE). Since the publication of NINDS–NIBIB criteria for the neuropathological diagnosis of CTE in 2016, and diagnostic refinements in 2021, hundreds of contact sport athletes and others have been diagnosed at postmortem examination with CTE. CTE has been reported in amateur and professional athletes, including a bull rider, boxers, wrestlers, and American, Canadian, and Australian rules football, rugby union, rugby league, soccer, and ice hockey players. The pathology of CTE is unique, characterized by a pathognomonic lesion consisting of a perivascular accumulation of neuronal phosphorylated tau (p-tau) variably alongside astrocytic aggregates at the depths of the cortical sulci, and a distinctive molecular structural configuration of p-tau fibrils that is unlike the changes observed with aging, Alzheimer’s disease, or any other tauopathy. Computational 3-D and finite element models predict the perivascular and sulcal location of p-tau pathology as these brain regions undergo the greatest mechanical deformation during head impact injury. Presently, CTE can be definitively diagnosed only by postmortem neuropathological examination; the corresponding clinical condition is known as traumatic encephalopathy syndrome (TES). Over 97% of CTE cases published have been reported in individuals with known exposure to repetitive head impacts (RHI), including concussions and nonconcussive impacts, most often experienced through participation in contact sports. While some suggest there is uncertainty whether a causal relationship exists between RHI and CTE, the preponderance of the evidence suggests a high likelihood of a causal relationship, a conclusion that is strengthened by the absence of any evidence for plausible alternative hypotheses. There is a robust dose–response relationship between CTE and years of American football play, a relationship that remains consistent even when rigorously accounting for selection bias. Furthermore, a recent study suggests that selection bias underestimates the observed risk. Here, we present the advances in the neuropathological diagnosis of CTE culminating with the development of the NINDS–NIBIB criteria, the multiple international studies that have used these criteria to report CTE in hundreds of contact sports players and others, and the evidence for a robust dose–response relationship between RHI and CTE.

Nearly 100 years ago, pathologist Harrison Martland introduced the term "punch-drunk” to describe a neurological condition in prizefighters [70, 71]. The boxers were described as “goofy” and “slug nutty,” and some progressed to “marked mental deterioration” and dementia [71] reviewed in Ref. [69]. The clinical syndrome was referred to by a variety of names, including "dementia pugilistica"[84] and "chronic traumatic encephalopathy" [13]. In 1957, Critchley observed that the neurobehavioral abnormalities often manifested years after the individual first began to box and continued to progress after the boxers retired, prompting him to suggest “chronic progressive traumatic encephalopathy” [27]. Regardless of the terms used in these early reports, they described a clinical syndrome that primarily affected boxers [71, 103]. Today, "chronic traumatic encephalopathy" or CTE refers exclusively to the tissue-based neuropathological diagnosis of CTE. The clinical syndrome associated with CTE pathology is known as traumatic encephalopathy syndrome (TES) [52]. Presently, CTE cannot be definitively diagnosed during life; the diagnosis is made after death by neuropathological examination. The criteria for the neuropathological diagnosis of CTE were established by two consensus conferences convened by the National Institute of Neurological Disorders and Stroke (NINDS) and National Institute of Biomedical Imaging and Bioengineering (NIBIB) [11, 74].

## Timeline of development of neuropathological criteria for the diagnosis of CTE

Case reports describing the neuropathology of CTE initially appeared in the 1950s and 1960s [24, 41, 72, 87, 99]. In 1973, Corsellis, Bruton, and Freeman-Browne described the neuropathological features in a series of 15 former male boxers, ranging in age from 57 to 91 years [25]. Corsellis et al. described macroscopic changes of cerebral atrophy, enlargement of the lateral and third ventricles, thinning of the corpus callosum, cavum septum pellucidum with fenestrations, and scarring of the cerebellar tonsils. Microscopically, using the histological methods available at the time, namely cresyl violet, von Braunmühl’s silver stain, and Kings amyloid stain, they reported sparse argyrophilic neurofibrillary tangles (NFTs) in the cerebral cortex and substantia nigra along with senile plaques in 20% of cases. Subsequent re-examination of Corsellis’ original series of boxers using beta-amyloid (Aβ) immunohistochemistry determined that 95% of cases had diffuse Aβ deposits [117]. Re-examination of the original cases in 2018 using hyperphosphorylated tau (p-tau) immunohistochemistry and applying 2016 NINDS–NIBIB consensus criteria found that 50% met criteria for CTE; however, prolonged formalin fixation and limited tissue availability might have affected the findings [39].

In 1991, Hof et al. described the neuropathology of a young autistic patient with repetitive head-banging behaviors using thioflavin and Gallyas silver methods, a silver technique more sensitive to argyrophilic inclusions than other silver methods [47, 60]. They described perivascular clusters of thioflavin and Gallyas positive NFTs and neurites at the depths of the sulci in the inferior temporal, entorhinal, and perirhinal cortices, in the absence of Aβ plaques [47]. They also quantitatively demonstrated the superficial distribution of the NFTs in layers II and III, a laminar predilection not found in Alzheimer’s disease (AD) [46]. In 1996, using AT8 immunohistochemistry, Geddes et al. described patchy, perivascular NFTs in the brain of a 23-year-old boxer [37]. They later compared the immunohistochemical findings of five young men, ranging from 23 to 28 years old, including the young boxer [36]. The men were exposed to RHI from head banging, poorly controlled epilepsy, rugby, and boxing. They described perivascular, p-tau-positive cortical NFTs and neuropil threads and noted that the pathology principally involved the depths of sulci [36]. No Aβ deposits were evident. Of the 21 age-matched controls they also examined, none showed a similar pathology.

Omalu et al. were the first to report the neuropathological findings of an American National Football League (NFL) player who had died at age 50 after experiencing cognitive impairment, mood changes, and parkinsonian symptoms [97]. The authors described mild, non-specific, tau pathology, consisting of sparse neocortical and locus coeruleus (LC) NFTs alongside diffuse Aβ plaques. In their second NFL case, sparse to frequent NFTs were found in the frontal and temporal cortices, diencephalon, and brainstem [96]. There was no mention of a perivascular or superficial pattern to the tau pathology, nor predilection for sulcal depths, features considered characteristic of CTE by Hof and Geddes. Although not highlighted by the authors, photomicrographs of the case showed clusters of perivascular p-tau pathology now considered pathognomonic for CTE [74]. P-tau pathology was also reported in a 40-year-old professional wrestler who died by suicide [98]. Sparse to frequent NFTs and neuropil threads in the cortex, subcortical ganglia, and brainstem nuclei were interpreted as CTE.

In 2009, McKee et al. detailed the neuropathological findings of two former boxers and a former NFL player and conducted a systematic review of all 48 previous neuropathologically verified cases of CTE in the world’s literature [75]. Using large format 50 µm sections and CP-13 and AT8 immunostaining to illustrate the regional p-tau pathology, the authors emphasized the distinctive p-tau pathology of CTE including cellular and regional abnormalities not previously detailed. The authors stressed the irregular, patchy distribution of NFTs, the prominent perivascular pattern, and the preferential involvement of cortical laminae II and III of the cortex. The authors also noted the predilection for the depths of the cortical sulci in the frontal, temporal, parietal, insular, and septal cortices with sparing of primary visual cortex. In addition, the authors described astrocytic p-tau inclusions, "astrocytic tangles" in subpial regions and around small blood vessels, and dot-like and spindle-shaped neurites. Dense NFTs, ghost tangles, and neurites were present in the hippocampus, entorhinal and transentorhinal cortices, amygdala, nucleus basalis of Meynert, hypothalamic nuclei, mammillary bodies, olfactory bulb, thalamus, substantia nigra pars compacta, dorsal and median raphe nuclei, and LC. The subcortical white matter, especially the subcortical U-fibers, corpus callosum, internal, external, and extreme capsules, fornix, and mammillothalamic tracts contained p-tau neurites, although the white matter was less affected than gray matter [75].

The neuropathological findings of 17 athletes ranging in age at death from 18 to 50 years were reported by Omalu et al. in 2011 [94]. CTE was diagnosed in seven of eight (88%) football players, two of four (50%) professional wrestlers, and one boxer. The p-tau pathology was described as sparse, moderate, or frequent, and NFTs ranged from band shaped or flame shaped to globose. Diffuse amyloid plaques were found in two cases. Omalu proposed pathological criteria for the diagnosis of CTE as four “emerging phenotypes" based on the presence or absence of NFTs and neuritic threads in the cerebral cortex, subcortical nuclei, and brainstem, with or without diffuse Aβ plaques. In the description of the phenotypes, there was no mention of specific morphological, cellular, or regional features to distinguish CTE p-tau pathology from AD, primary age-related tauopathy (PART), progressive supranuclear palsy (PSP), corticobasal degeneration (CBD), or other tauopathies.

The first military veteran with CTE was reported in 2011 [95]. Omalu et al. described a 27-year-old Iraqi war veteran who experienced combat and was exposed to improvised explosive device blasts. Neuropathological examination revealed multifocal, cortical, and subcortical NFTs and neuritic threads, accentuated in the frontal cortex and at the depths of the sulci. In 2012, Goldstein et al. described the neuropathological features found in four male military veterans with known blast exposure or concussive injury (ages 22–45 years; mean, 32.3 years), three amateur American football players and a professional wrestler (ages 17–27 years; mean, 20.8 years), and four normal controls without a history of RHI (ages 18–24 years; mean, 20.5 years) [40]. In the young military veterans and athletes, perivascular foci of p-tau NFTs were found in the frontal cortices with a predilection for sulcal depths. None of the controls demonstrated p-tau pathology.

In 2013, McKee et al. reported the findings in a series of 85 subjects, all but one male, ranging in age from 17 to 98 years, mean 59.5 years, with a history of repetitive head impacts (RHI) from American football, ice hockey, boxing, military service, and head-banging behaviors, and compared them to 18 age- and gender-matched controls without a history of RHI [79]. They described a distinctive pattern of p-tau pathology in 68 men that they considered diagnostic for CTE and proposed the McKee criteria for the pathological diagnosis of CTE. The diagnosis of CTE required: (1) the presence of perivascular foci of p-tau NFTs and astrocytic tangles; (2) an irregular cortical distribution of p-tau NFTs and astrocytic tangles with a predilection for the depth of cerebral sulci; (3) clusters of subpial and periventricular astrocytic tangles in the cerebral cortex, diencephalon, basal ganglia, and brainstem; and (4) NFTs in the cerebral cortex located preferentially in the superficial layers.

Among those diagnosed with CTE, the spectrum of p-tau pathology ranged in severity from sparse perivascular foci of NFTs in the cortex, usually the frontal cortex, to a severe, widespread tauopathy affecting the medial temporal lobe, thalamus, hypothalamus, mammillary bodies, basal ganglia, brainstem, cerebellum, and white matter tracts. The p-tau pathology followed an ordered, hierarchical progression of severity, prompting the authors to propose a staging scheme: McKee stages I–IV. Stage I CTE was characterized by isolated cortical clusters of perivascular NFTs, astrocytic tangles, and dot-like neurites (i.e., “CTE lesions”), most prominent at the depths of the sulci, typically affecting the dorsolateral frontal cortices. In stage II CTE, multiple CTE lesions were found in the frontal, temporal, and parietal cortices with sparse NFTs in the superficial cortical layers, LC, and nucleus basalis of Meynert. Stage III CTE was characterized by multiple focal cortical CTE lesions, NFTs in the superficial cortical laminae and NFTs in widespread cortical areas, hippocampus, entorhinal cortex, amygdala, nucleus basalis of Meynert, substantia nigra, dorsal and median raphe, LC, and olfactory bulbs. In stage IV CTE, NFTs and CTE lesions were densely distributed throughout the cerebral cortex, with severe neurofibrillary degeneration and ghost tangles in the medial temporal lobe structures, and NFTs in the thalamus, mammillary bodies, nucleus basalis of Meynert, substantia nigra, dorsal and median raphe, LC, basis pontis and cerebellar dentate nucleus, often with neuronal loss and gliosis. The p-tau pathology consisted of three-repeat (3R) and four-repeat (4R) tau; the astrocytic tangles were predominantly 4R tau.

The stages of CTE were associated with progressive increases in macroscopic abnormalities: typically mild dilatation of the frontal horn of the lateral ventricles or third ventricle with cavum septum pellucidum in stages I and II, evolving to mild cerebral atrophy, depigmentation of the LC and substantia nigra in stage III, and severe atrophy of the frontal, temporal, and medial temporal lobes, a sharply concave contour of the third ventricle, cavum septum pellucidum and septal perforations, thalamic atrophy, a sharply convex contour of the medial thalamus ("thalamic notch"), thinning of the hypothalamic floor, atrophy of mammillary bodies, depigmentation of the LC and substantia nigra, and thinning of the posterior corpus callosum in stage IV.

Comorbid neurodegenerative disease was present in 25 of the 68 CTE cases (36.8%) including AD, Lewy body disease (LBD), frontotemporal lobar degeneration (FTLD)-TAR DNA-binding protein (TDP), progressive supranuclear palsy (PSP) and Pick’s disease. Aβ deposition was found in 44.1%, primarily as diffuse plaques, and was significantly associated with age at death.

Although not well characterized at the time, McKee et al. described a form of astrocytic p-tau pathology in CTE, now termed age-related tau astrogliopathy (ARTAG), consisting of 4R immunoreactive ‘thorn-shaped astrocytes’ (TSA) in the subpial, periventricular, and perivascular white matter, and less frequently in the gray matter. TSA had been previously described in the aging brain [48, 59, 61], although at the time of the 2013 publication, it was unclear whether the subpial and periventricular TSA and astrocytic tangles found in CTE were unique to CTE or a form of ARTAG. Subsequently, an international group of neuropathologists led by Gabor Kovacs defined the many p-tau astrocytic morphologies of ARTAG, resulting in improved discrimination between pathologies considered typical of ARTAG and those specific to CTE [58].

In 2013, Hazrati et al. reported the clinical and pathological findings in six former Canadian Football League players [44]. Three of the six (50%) cases had neuropathological findings diagnostic for CTE, defined as NFTs and astrocytic tangles in a patchy, perivascular distribution, localized to the depths of sulci, subpial areas, and the superficial cortical layers (layers II/III). In 2015, Bieniek et al. reviewed available medical records of 1,721 men in the Mayo Clinic neurodegenerative disease-focused brain bank for evidence of a history of traumatic brain injury (TBI) or participation in contact sports [12]. They identified 66 cases with a documented history of sports exposure and subsequently processed frontal and parietal cortical tissue samples for p-tau immunohistochemistry. Tissue samples in 198 age- and disease-matched men and women without contact sports exposure were similarly processed. Of the 66 men with exposure to contact sports, 21 (32%) had p-tau pathology consistent with CTE characterized by perivascular foci of TSA, NFTs, and threads at the depths of cortical sulci. Seven cases were classified as CTE stage I, seven as CTE stage II, five as CTE stage III, and two as CTE stage IV. The 198 controls without contact sports exposure showed no CTE pathology, including 33 individuals with single-incident TBI. The authors also drew attention to ARTAG pathology, most marked in two boxers, and commented that ARTAG must be considered in the differential diagnosis of CTE, especially in those with advanced age.

**Fig. 1:**
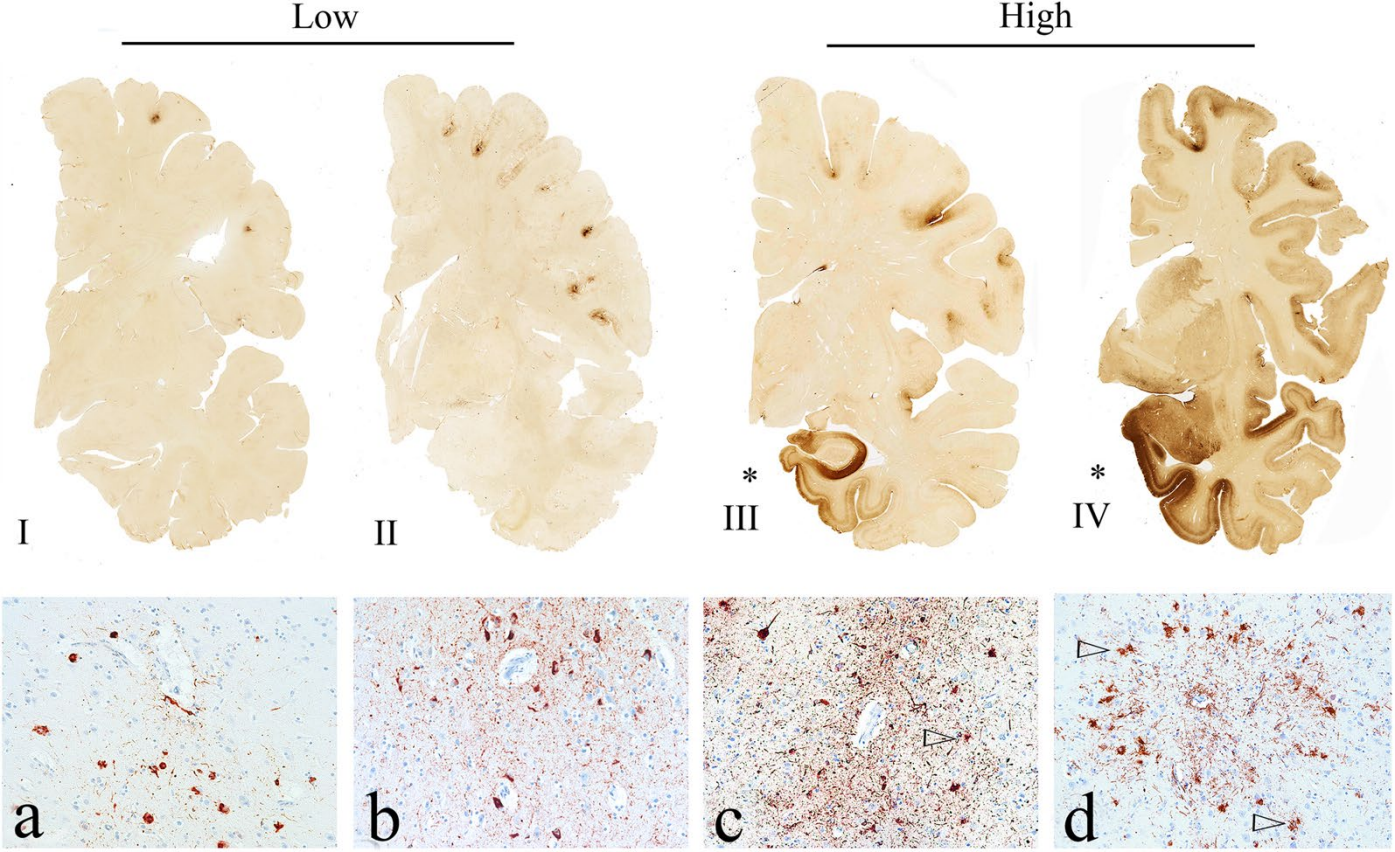
The pathognomonic lesion of CTE and the staging schemes of pathological severity (adapted with permission from [83]). Representative images of p-tau pathology at Low and High chronic traumatic encephalopathy (CTE) pathological stage using the abbreviated staging scheme recommended by the second NINDS/NIBIB consensus panel (low–high) [11] and the McKee staging scheme (I–IV) [4, 79]. Low CTE is characterized by p-tau pathology restricted to focal cortical lesions. High CTE shows widespread p-tau pathology in the medial temporal lobe structures and diencephalon in addition to focal cortical lesions. McKee Stage I CTE is characterized by one or two isolated CTE lesions at the depths of the cortical sulci. In stage II, three or more cortical CTE lesions are found. In stage III CTE, there are multiple CTE lesions and diffuse NFTs in the medial temporal lobe. In stage IV CTE, CTE lesions and NFTs are widely distributed throughout the cerebral cortex, diencephalon, and brainstem. Top row: hemispheric 50-µm tissue sections immunostained with CP-13, directed against phosphoserine 202 of tau (courtesy of Peter Davies, Ph.D., Feinstein Institute for Medical Research; 1:200); positive p-tau immunostaining appears dark brown. Bottom row: 10-µm paraffin-embedded tissue sections immunostained for phosphorylated tau (AT8) (Pierce Endogen). Positive p-tau immunostaining appears dark red, hematoxylin counterstain. **I** A 26-year-old former college football player with stage I CTE (Low). Two perivascular p-tau CTE lesions are evident at the sulcal depths of the frontal cortex; there is no neurofibrillary degeneration in the medial temporal lobe. **II** A 49-year-old former NFL player with stage II CTE (Low). There are multiple perivascular p-tau CTE lesions at depths of sulci of the frontal cortex; there is no neurofibrillary degeneration in the amygdala or entorhinal cortex. **III** A 53-year-old former NFL player with stage III CTE (High). There are multiple CTE lesions in the frontal cortex and insula; there is diffuse neurofibrillary degeneration of hippocampus and entorhinal cortex (asterisk). **IV** A 62-year-old former NFL player with stage IV CTE (High). There are multiple CTE lesions in the cerebral cortex with widespread neurofibrillary degeneration. There is also extensive neurofibrillary degeneration of the amygdala and entorhinal cortex (asterisk). **a** Pathognomonic CTE lesion in stage I CTE. AT8 immunopositive neurofibrillary tangles, dot-like and threadlike neurites encircle a small blood vessel. **b** Pathognomonic CTE lesion in stage II CTE. A cluster of AT8 immunopositive neurofibrillary tangles and dense dot-like neurites surround several small blood vessels, **c** pathognomonic CTE lesion in stage III CTE. A large collection of AT8 immunopositive neurofibrillary tangles and dense dot-like neurites enclose several small blood vessels. With increasing age, AT8 immunoreactive astrocytes are increasingly evident within the pathognomonic lesion (open triangle). **d** Pathognomonic CTE lesion in stage IV CTE. A large accumulation of AT8 immunopositive neurofibrillary tangles, most of them ghost tangles, encompass several small blood vessels. With increasing age, AT8 immunoreactive astrocytes are increasingly prominent (open triangles) and there may be evidence of neuronal loss. **a–d** All magnification × 200. *P-tau* phosphorylated tau, *CTE* chronic traumatic encephalopathy, *NFL* National Football League

In 2014, the NINDS–NIBIB organized a consensus meeting to establish the neuropathological criteria for the diagnosis of CTE using the McKee criteria for CTE as a starting point [74, 79]. By the time the consensus panel met in November 2015, ARTAG was recognized as an age-related p-tau pathology characterized by multiple patterns of astrocytic p-tau, including subpial TSA that are commonly found in CTE. Several members of the consensus panel were involved in developing the harmonization criteria for ARTAG [58]. Recognizing that ARTAG pathology is frequently found in CTE but is non-diagnostic, the criteria for the diagnosis of CTE used by the consensus panel were modified to include: perivascular foci of p-tau NFTs and astrocytic tangles in the cortex; irregular clusters of p-tau NFTs and astrocytic tangles found preferentially at the sulcal depths; NFTs in the cerebral cortex located primarily in the superficial layers. Subpial TSA, or ARTAG, were considered only supportive, non-diagnostic, features of CTE. With these modified CTE criteria, a panel of expert neuropathologists evaluated 25 cases of various tauopathies blinded to all clinical, demographic, and gross neuropathological information. The 25 cases included 10 cases of suspected CTE, and 15 other cases, including AD, PSP, PART, argyrophilic grain disease, CBD, and parkinsonism dementia complex of Guam. A single laboratory processed all cases uniformly and the resulting slides were scanned into digital images that were provided to neuropathologists blinded to all other information. The neuropathologists submitted their independent evaluations prior to meeting in person. The panel found that the criteria reliably distinguished CTE from the other tauopathies and established the preliminary NINDS–NIBIB criteria for the pathological diagnosis of CTE. Importantly, the panel defined a *pathognomonic* lesion of CTE as the "accumulation of abnormally phosphorylated tau in neurons and astroglia distributed around small blood vessels at the depths of cortical sulci and in an irregular pattern." (Fig. 1) They further observed that the p-tau neurites in CTE were often dot-like, that TDP-43-immunoreactive inclusions in CTE were distinctive, and the pattern of hippocampal neurofibrillary degeneration was unlike AD. They also made recommendations for the diagnosis and evaluation process of potential CTE cases [74].

**Fig. 2:**
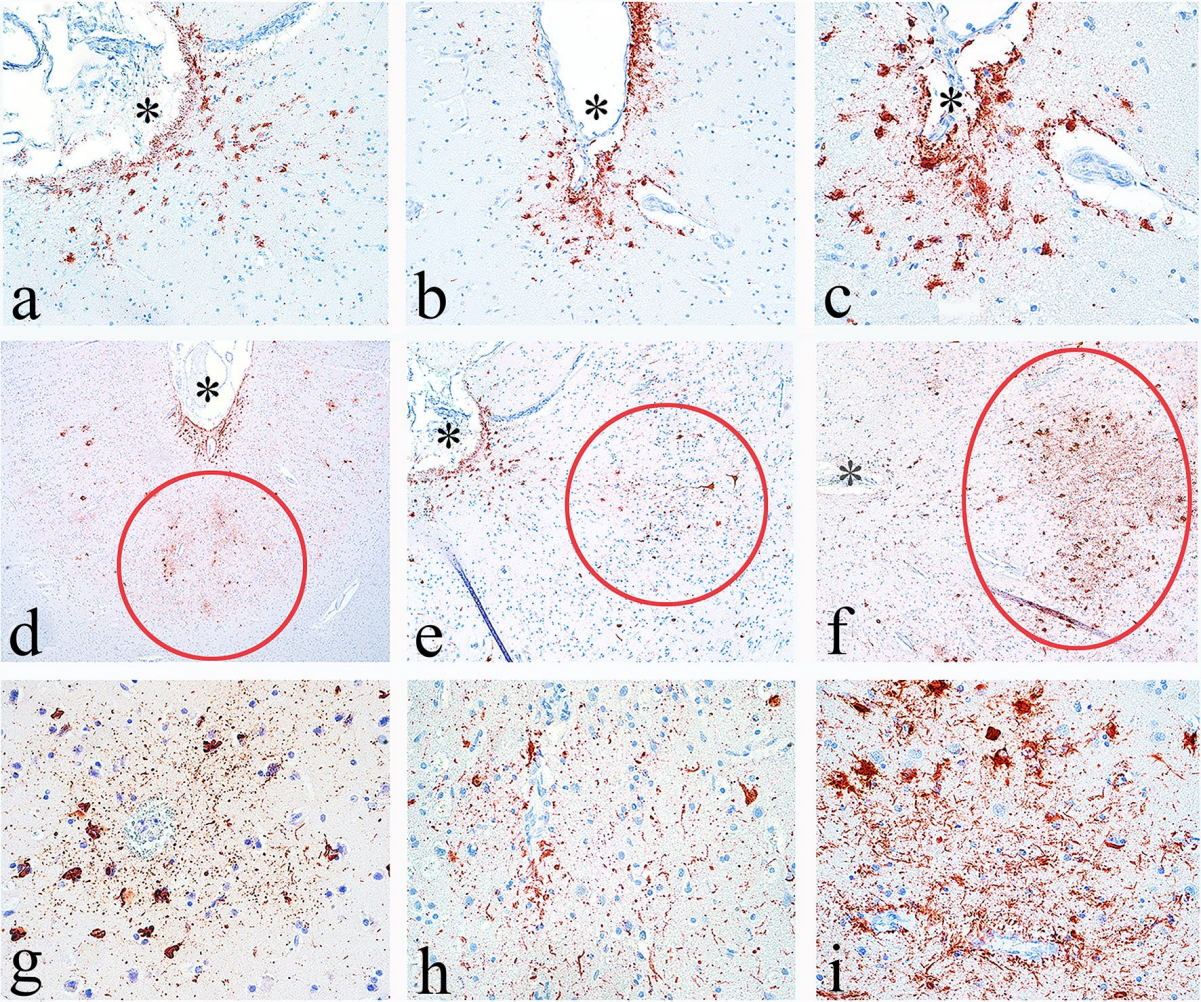
Differential diagnosis between mild CTE and ARTAG. 10-µm paraffin-embedded tissue sections immunostained for phosphorylated tau (AT8) (Pierce Endogen). Positive p-tau immunostaining appears dark red, hematoxylin counterstain. **a–c** p-tau-immunoreactive thorn-shaped astrocytes are present at the glial limitans at the depths of the sulcus, a form of ARTAG (**a, b)** magnification × 200, **c** magnification × 400). The depth of the sulcus is marked by an asterisk. **d–f** Clusters of p-tau neurons and dot-like neurites surrounding small blood vessels in deep cortical laminae at the depth of the sulci are representative of the diagnostic CTE lesion, all magnifications × 40. The diagnostic lesions are indicated by red circles and located deeper than subpial ARTAG, marked by an asterisks. **g–i** CTE lesions consist of p-tau-immunoreactive neurons, dot-like neurites, and variably astrocytes, surrounding small blood vessels, all magnifications × 200

During the years 2013–2016, when ARTAG was emerging as an age-related tau pathology but not fully recognized by all neuropathologists, several publications reported the prevalence of CTE in brain bank collections and autopsy series that might have misinterpreted ARTAG for diagnostic CTE pathology (Fig. 2) [66, 92, 102]. In 2015, Ling et al. screened a consecutive series of brain donations to the Queen Square Brain Bank for Neurological Disorders for CTE using the proposed McKee criteria for CTE [66, 79]. Their total sample included 221 elderly individuals with neuropathological evidence of a neurodegenerative disease including PSP, LBD, multiple system atrophy, CBD, FTLD, and AD, and 47 controls (> 60 years of age). They identified 32 (11.9%) cases as having neuropathology consistent with CTE, mean age at death 81.0 years, including 13 females (40.6%). The prevalence of CTE in controls (12.8%) was essentially the same as in those with neurodegenerative disorders (11.8%). In hindsight, although we have not had the opportunity to review the histology, it is possible the authors might have considered subpial ARTAG as diagnostic of CTE in some of their cases, as the images they provide depicting characteristic CTE pathology (e.g., Ling et al. [66], Fig. 1) appear to show purely astrocytic perivascular p-tau pathology that would not meet current NINDS–NIBIB diagnostic criteria for CTE [11, 74]. In addition, 15 of their 32 CTE cases (47%) had diagnostic pathology restricted to the midbrain. Isolated midbrain p-tau pathology would not meet current criteria for CTE; at a minimum, the diagnosis of CTE requires at least one pathognomonic *cortical* lesion [11]. In addition, the advanced age (81.0 years) and female gender (47%) of their cases would be unusual for early-stage CTE (all cases were CTE stage I or II), but typical for ARTAG. Nevertheless, postmortem interviews with the next of kin indicated that 93.8% of their CTE cases had some form of exposure to RHI. Noy et al. prospectively examined 111 brains in a non-selected community-based neuropathology service in Winnipeg, Manitoba, Canada, for the presence of CTE pathology [92]. They excluded brains from individuals over age 60 or with any histological evidence of AD. They identified five cases that met full criteria for CTE based on the NINDS–NIBIB consensus criteria: three cases of stage I and two cases of stage II CTE. They also identified 34 cases (30.6%) that showed "CTE < 1", a category defined as small p-tau deposits in perivascular regions at the depths of cortical sulci. It is unclear how many of their CTE < 1 cases might have represented ARTAG. Among a separate group, they also identified four cases that met full criteria for CTE. All four cases with CTE had a history of head trauma, although detailed sports histories were not available. Puvenna et al. examined 6 pathologically verified cases of CTE (mean age at death, 73.3 years), 6 age-matched control samples, and 19 surgically resected brain specimens from individuals with temporal lobe epilepsy (TLE) (ranging in age from 4 months to 58 years; mean 27.6 years) [102]. Puvenna et al. reported that p-tau pathology in the TLE specimens was identical to that in the CTE specimens. Although we were not able to examine the TLE brain tissue, the CTE brain specimens originated from the Boston University Understanding Neurological Injury and Traumatic Encephalopathy (UNITE) brain bank, and we were unable to appreciate any diagnostic CTE pathology in the representative figures supplied in the manuscript. Later studies would report a non-specific increase in p-tau pathology in surgical specimens from epilepsy patients, unrelated to CTE [109].

In 2016, the consensus panel met again to review and refine the preliminary pathological criteria for CTE provided by the first NINDS–NIBIB consensus conference using a second blinded sample of CTE cases representing all severities of disease: mild to severe [11]. The panel sought to further distinguish CTE from ARTAG and from PART [26], to address the minimum threshold for diagnosis, and to determine whether the McKee staging scheme was reliable using only a limited number of paraffin-embedded slides instead of large format 50 µm sections [79]. Eight neuropathologists evaluated 27 cases of tauopathies (17 CTE) and were highly accurate in CTE diagnosis using the preliminary NINDS–NIBIB criteria. In the first round, blinded to clinical and demographic data and gross neuropathological features, 88% of the responses correctly indicated a diagnosis of CTE, which rose to 97% after the clinical data and gross neuropathological features were supplied. Generalized estimating equation analyses showed a statistically significant association, with large effect sizes, between the raters’ assessment and the cases submitted as CTE for both the blinded [odds ratio (OR) 72, 95% confidence interval (CI) 19–267] and unblinded rounds (OR 257, 95% CI 64–1559). The panel concluded by refining the definition of the pathognomonic lesion to emphasize that the perivascular p-tau aggregates necessarily involve neurons, with or without p-tau in astrocytes, and are found in deeper cortical layers than the subpial and superficial regions (Fig. 2, Tables 1, 2). In addition, the panel confirmed that subpial TSA and purely astrocytic perivascular p-tau pathology represented ARTAG and did not meet the minimum criteria for CTE.

**Table 1:** Recommended protocol for evaluation for CTE [11, 74]

1. The diagnosis of CTE is made using 10 mm-thick paraffin-embedded tissue sections from the following brain regions immunostained for p-tau
(1) Middle frontal gyrus
(2) Superior and middle temporal gyri
(3) Inferior parietal lobule
(4) Hippocampus
(5) Amygdala
(6) Basal ganglia
(7) Thalamus
(8) Midbrain with substantia nigra
(9) Pons with locus coeruleus
(10) Medulla oblongata
(11) Cerebellar cortex with dentate nucleus
2. Recommended p-tau antibodies: AT8, CP-13, PHF-1 and 4R tau
3. When CTE is suspected but not evident in the initial slides, additional cortical sampling is recommended. It is recommended that the resampled tissue capture 4–8 cortical sulci (preferably bilateral) from the superior frontal, dorsolateral superior frontal, superior middle temporal, and/or inferior temporal gyri
4. In cases with only subpial TSA, superficial laminar NFT or non-specific sulcal p-tau pathology, the case is not diagnostic for CTE, and resampling is recommended
5. If a CTE lesion is detected with resampling, the case is diagnosed as CTE. If no CTE lesions are identified with resampling, the case is considered not diagnostic for CTE

**Table 2:** NINDS-NIBIB criteria for the pathological diagnosis of CTE [11]

*Minimum threshold for diagnosis of CTE*
The presence of a single pathognomonic lesion in the cortex (CTE lesion). The pathognomonic CTE lesion consists of p-tau aggregates in neurons, with or without glial p-tau, at the depth of a cortical sulcus around a small blood vessel. The CTE lesion is found in deeper cortical layers of the sulcus and not restricted to subpial and superficial regions
*Supportive p-tau-related features of CTE*
1. Neurofibrillary tangles (NFT) principally affecting superficial layers (layers II–III), often prominent in the temporal lobe
2. In the hippocampus, pretangles and NFT in CA2; pretangles and prominent proximal dendritic swellings in CA4
3. NFT and astrocytic p-tau aggregates in the thalamus, hypothalamus (including mammillary bodies) amygdala, nucleus basalis of Meynert, substantia nigra raphe nuclei, and locus coeruleus
4. Dot-like neurites, in addition to some threadlike neurites
*Aging-related tau astrogliopathy (ARTAG)* including thorn-shaped astrocytes (TSA) in the subpial region may be present but are non-diagnostic

At this second consensus meeting, applying the McKee staging system to the limited series of paraffin-embedded slides proved to be inconsistent. P-tau pathology in CTE is patchy and widely dispersed in mild cases and might not be captured using restricted sampling. To aid in the assessment of CTE pathological severity when only a limited number of paraffin-embedded slides are available, the panel proposed a simplified working protocol. The algorithm considers cases as diagnostic for CTE if a single pathognomonic lesion is present, then classifies the case as either “Low CTE” or “High CTE,” depending on whether NFTs are present in the thalamus, mammillary bodies, hippocampus, amygdala, and entorhinal cortex. The designation “Low CTE” roughly equates to McKee CTE stages I and II, and “High CTE” to McKee CTE stages III and IV (Fig. 1, Table 3). The designations high and low CTE were not intended to replace the McKee staging scheme but were designed to facilitate evaluating disease severity by neuropathologists in routine practice.

**Table 3:** Systems to evaluate pathological severity in CTE

I. NINDS-NIBIB: low and high CTE [11], a practical staging system using only a limited number of regional p-tau immunostained slides
1. In the cortical region containing the most prominent CTE lesion, NFT in the bank or crest of the gyrus each count as 1 point, NFT in the superficial cortical laminae also count as 1 point
2. NFT in CA1 and CA4 of the hippocampus, entorhinal cortex, amygdala, thalamus, mammillary body, and cerebellar dentate nucleus also count as 1 point each, for a total of 10 possible points per case
Low CTE: cases with 1–4 points are considered mild cases of CTE, roughly equivalent to McKee stages I–II
High CTE: cases with 5 or more points are considered severe cases of CTE; roughly equivalent to McKee stages III-IV
II. McKee Staging Scheme: CTE I–IV [4, 79], a four-tiered staging system using a comprehensive set of regional p-tau immunostained slides or large format whole mount 50 µm sections
Stage I: one or two isolated pathognomonic CTE lesions in the cortex, NFT in LC
Stage II: three or more CTE lesions in multiple cortical regions. NFT in LC and SN
Stage III: three or more CTE lesions in multiple cortical regions. NFT in the hippocampus, entorhinal cortex, and amygdala. NFT in thalamus, mammillary body, nucleus basalis of Meynert, SN, raphe nuclei, and LC
Stage IV: three or more CTE lesions in multiple cortical regions. Marked NFT in the hippocampus, entorhinal cortex, and amygdala. Marked NFT in the thalamus, mammillary body, nucleus basalis of Meynert, SN, raphe nuclei, and LC. NFT in the cerebellar dentate nucleus, basis pontis, and spinal cord

It is notable that while the pathognomonic lesion of CTE is cortical, brainstem p-tau pathology is an essential aspect of CTE. The first and second consensus panels recommended that the midbrain, pons, and medulla be sampled and evaluated [11, 74], as brainstem p-tau pathology is a supportive feature of CTE. In CTE, p-tau NFT are often found in the isodendritic core (nucleus basalis of Meynert, raphe nuclei, substantia nigra, and LC), with the LC affected at all stages, including stage I.

## Retrospective cohort studies using NINDS–NIBIB criteria for the pathological diagnosis of CTE

After publication of the preliminary NINDS–NIBIB criteria in 2016 [74], multiple academic neuropathology centers and brain banks in England, Scotland, Ireland, Brazil, Canada, Australia, and the USA reported CTE in the brains of male athletes, including boxers [67, 120], soccer [42, 63, 68], rugby union [63, 113], rugby league [16], ice hockey [105], a bull rider [53], and large cohorts of American football players [4, 83] (Table 4). When the findings of the second consensus conference on CTE were published in 2021, there was further expansion in the number of international academic neuropathology centers interested in CTE and CTE research. Investigators analyzed community populations [100], international routine neuropathology series and brain banks [73, 114], and a convenience sample of consecutive autopsies of military personnel [101], for the presence of CTE.

**Table 4:**
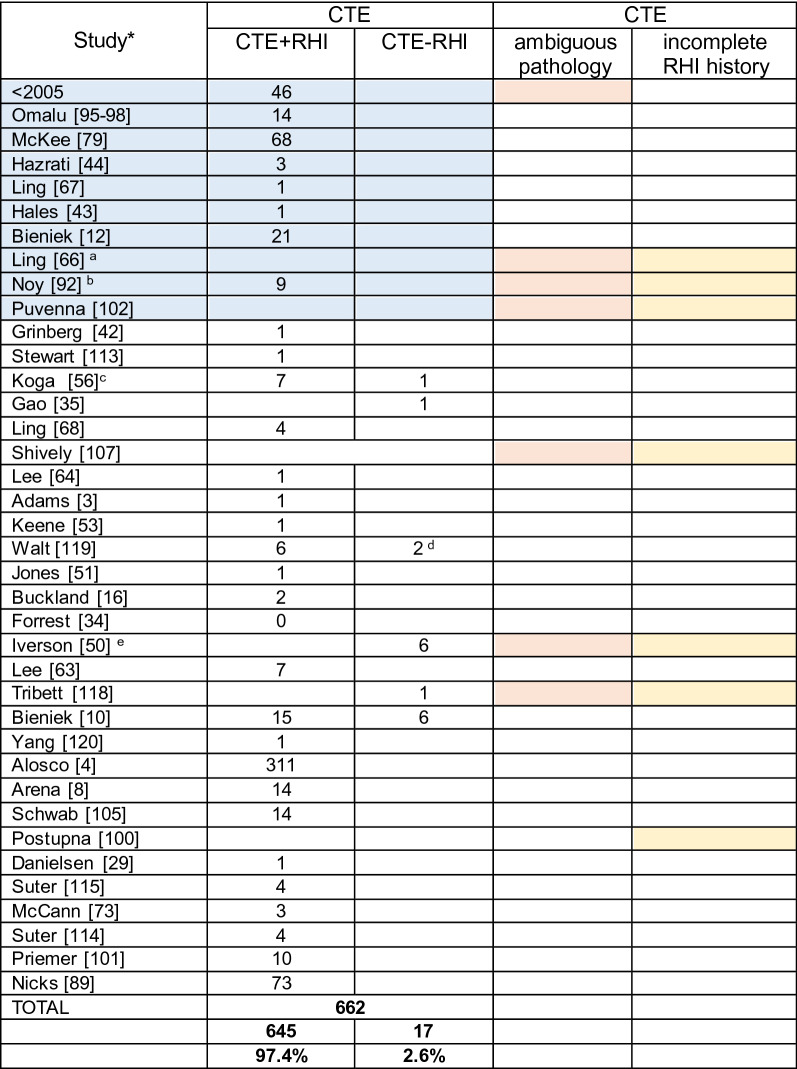
Cases with CTE Pathology and History of RHI

Blue shading indicates studies performed prior to publication of NINDS–NIBIB criteria [11, 74]

Pink shading indicates that there is some ambiguity whether all the cases reported met NINDS–NIBIB criteria for CTE, either because the report preceded the publication of the NINDS–NIBIB criteria or a pathognomonic lesion of CTE was not described

Yellow shading indicates that determination of RHI status was not performed or was incomplete

The numbers in the Table 4 represent all cases of CTE reported in the literature that included neuropathological analysis. Despite comprehensive search, it is possible that some case reports were missed. Also, if a study team published multiple case series, the number was adjusted to represent only additional cases, although this was an assumption, and the numbers might be inaccurate. Other caveats include:

*CTE* chronic traumatic encephalopathy, *RHI* repetitive head impacts, *ARTAG* age-related tau astrogliopathy, *NSC* non-specific changes

a. Ling et al. reported 32 cases of CTE, "most" with a history of TBI. We did not include these cases in the table because the cases were reported prior the publication of the NINDS–NIBIB criteria, and it is possible that some of the cases represented ARTAG (including the 15 (47%) based solely on midbrain pathology) [66]. In addition, Ling et al. performed no standardized assessment of RHI exposure

b. Noy et al. reported 5 CTE cases in their autopsy series plus 4 other cases of CTE (*n* = 9) and 34 cases with " < 1 CTE pathology" [92]. The 34 cases with < 1 CTE pathology were not included in the table as it is uncertain whether they met diagnostic criteria for CTE. In addition, the RHI history was incomplete

c. Koga reported eight cases that met diagnostic criteria for CTE, four with documented contact sports, and three with multiple falls, the exposure history for the eighth case was unknown [56]

d. In Walt et al., history of RHI was unknown in two cases, although one had a TBI from a fall with hospitalization, and the other had multiple falls secondary to ALS [119]

e. The cases from Iverson et al. were included in the table, although the RHI history was incomplete and there remains uncertainty whether the pathology was diagnostic for CTE [78]

### American football

Mez et al. reported the clinical and pathological features of a sequential brain bank series of 202 American football players [83]. Using the NINDS–NIBIB criteria for the diagnosis of CTE. CTE was neuropathologically diagnosed in 177 athletes [87%, median age at death, 67 years (IQR 52, 77); mean years of football participation, 15.1 years (5.2 SD)], including 0 of 2 pre-high school (0%), 3 of 14 high school (21%), 48 of 53 college (91%), 9 of 14 semi-professional (64%), 7 of 8 Canadian Football League (88%) and 110 of 111 NFL players (99%). The median age at death for American football players with mild CTE pathology (stages I and II) was 44 years (IQR 29, 64), and for participants with severe CTE pathology (stages III and IV) 71 years (IQR 64, 79). In all 177 cases, pathognomonic lesions of CTE were found in the cortex. Amyloid-β was present in a subset of participants at all stages of CTE pathology, predominantly as diffuse amyloid-β plaques. In stage IV CTE, amyloid-β deposition occurred in 52 cases (91%), TDP-43 deposition in 47 (83%), and α-synuclein deposition in 23 (40%) of cases.

**Fig. 3:**
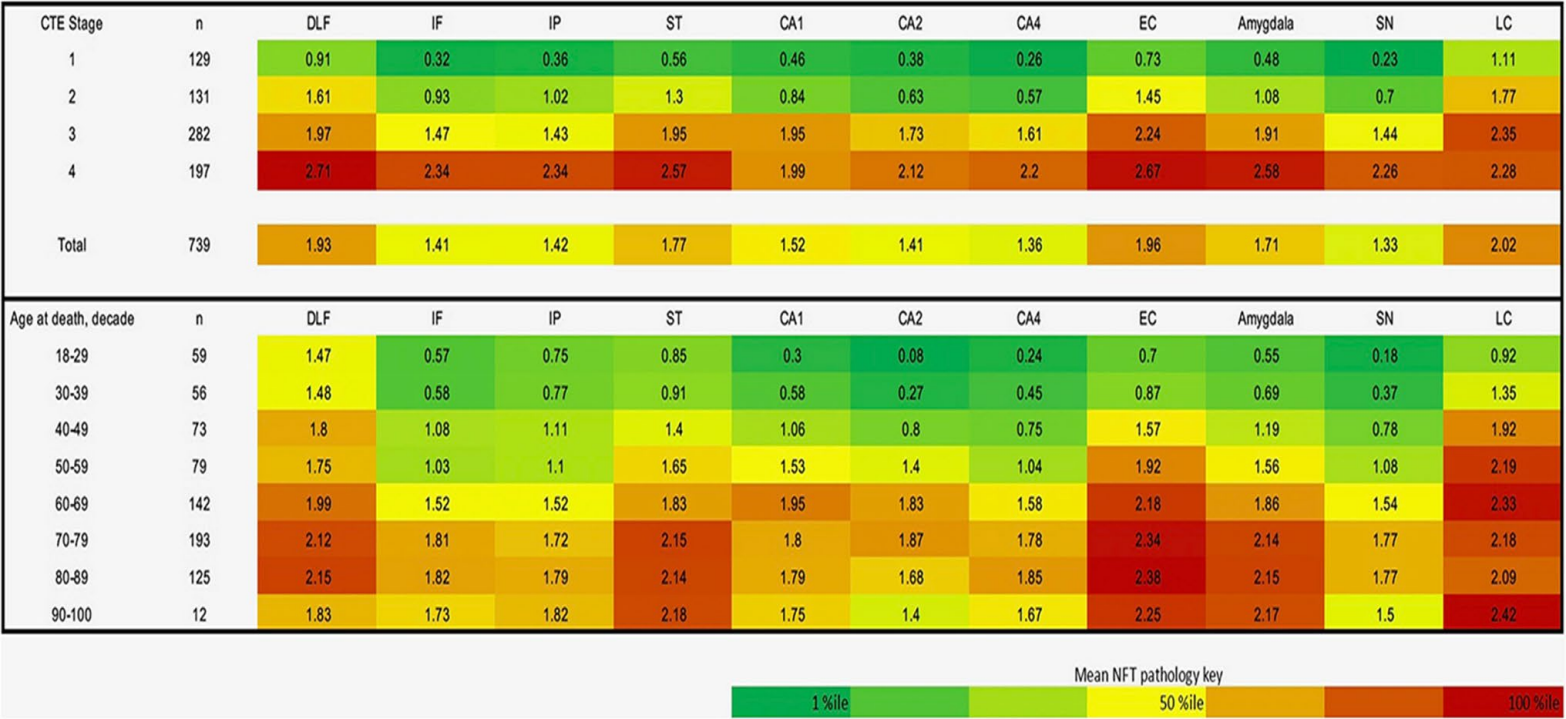
Regional progression of semi-quantitative p-tau density in CTE by stage and age (*n* = 739). Top: among 739 cases of neuropathologically verified CTE cases in the UNITE brain bank, there is a hierarchical increase in p-tau pathology across CTE stages I–IV. In stage I CTE, p-tau pathology is most dense in CTE lesions in the dorsolateral frontal cortex and NFT are found in the locus coeruleus (LC). In CTE stage II, there are increasing densities of p-tau pathology in the cortex, especially dorsolateral frontal and temporal lobes, entorhinal cortex, amygdala, and LC. In CTE stage III, there are further increases in p-tau pathology in the same regions with expansion to CA1 hippocampus and substantia nigra. In stage IV, p-tau densities continue to increase with highest densities in dorsolateral frontal and superior temporal cortices, entorhinal cortex, amygdala, and LC. Bottom: across decade at death, p-tau pathology in CTE shows a similar pattern of progressive regional involvement with highest densities of p-tau pathology in the dorsolateral frontal and superior temporal cortices, entorhinal cortex, amygdala, and LC. The most common locations of pathognomonic CTE lesions differ slightly from the areas of highest p-tau density. In a preliminary analysis of a subset of 52 of the 739 cases, pathognomonic lesions were most often found in the dorsolateral (56%, often multiple) and superior frontal cortices (56%), followed by Rolandic (33%) and inferior parietal (33%), septal (17%), insula and superior temporal (each 12%), hippocampus and entorhinal (each 8%), inferior frontal (6%) and temporal pole (< 1%). While most pathognomonic lesions involved the neocortex, they were also found in hippocampus, amygdala and entorhinal cortex. Similarly, while most pathognomonic lesions were found at the depths of the neocortical sulci, occasionally they were present in areas with no sulci, e.g., along gyral banks, in hippocampal subfields, and in the amygdala. A superficial distribution of NFT is often most prominent in the temporal lobe. *DLF* dorsolateral frontal, *IF* inferior frontal, *IP* inferior parietal, *ST* superior temporal, *CA1* CA1 hippocampus, *CA2* CA2 hippocampus, *CA4* CA4 hippocampus, *EC* entorhinal cortex, *SN* substantia nigra, *LC* locus coeruleus. In each region, dark green is the 1st percentile, light green is the 25th percentile, yellow is the 50th percentile, orange is the 75th percentile and dark red is the 100th percentile. The color scale is based on the distribution of all values, not by each individual stage. Values represent means of p-tau pathology among participants in each stage or age decade at death

The largest series of American football players with neuropathologically diagnosed CTE was published by Alosco et al. [4]. In this series of 366 male brain donors, the authors tested the utility of the McKee CTE staging scheme by examining the relationship between CTE stage and semi-quantitative and quantitative assessments of regional p-tau pathology, age at death, dementia, and years of American football play. Spearman’s rho correlations showed that higher CTE stage was associated with higher scores on all semi-quantitative and quantitative assessments of p-tau severity and density (*p* < 0.001). The severity and distribution of CTE p-tau followed an age-dependent progression and CTE stage was independently associated with increased odds for dementia (*p* < 0.001). K-medoids cluster analysis of the semi-quantitative scales of p-tau across 14 regions identified five clusters of p-tau that conformed to increasing McKee CTE stage (with stage IV having two slightly different clusters), age at death, dementia, and years of American football play. P-tau pathology was most prevalent in five regions: DLF, superior temporal cortex, entorhinal cortex, amygdala, and LC, with CTE in the youngest brain donors and lowest CTE stage restricted to DLF and LC. These findings support a progressive, hierarchical regional distribution of p-tau in CTE. Recent analysis of 739 neuropathologically verified cases of CTE from the UNITE brain bank confirmed the hierarchical progression of p-tau pathology across CTE stage and age decade at death (Fig. 3).

### Soccer

CTE was first reported in a soccer player in the case of a 29-year-old semi-professional player with CTE stage II and amyotrophic lateral sclerosis (ALS) [76]. Subsequently, Hales and Grinberg independently reported the neuropathological findings of two former professional soccer players who died in their 80s with severe dementia. Late-stage CTE, TDP-43 proteinopathy, and varying degrees of AD pathology were found in both; one also had hippocampal sclerosis [42, 43]. In 2017, Ling et al. reported a series of six retired professional soccer players with dementia who were followed longitudinally and consented to postmortem brain examination [68]. Using the NINDS-NIBIB consensus criteria for CTE, four of six were diagnosed with CTE. In all four, there was also evidence of AD and TDP-43 pathology.

### Rugby union and rugby league

After the initial report of CTE in a young rugby player [36], Stewart et al. found CTE^1^ in a 57-year-old rugby union player [113], then expanded their findings in a series of 11 former soccer and rugby union players with dementia and comprehensive neuropathological examinations [63]. The authors considered the neuropathological findings along with non-blinded, retrospective clinical histories to derive an integrated clinicopathological diagnosis for each case. Using NINDS–NIBIB neuropathological criteria, CTE was found in five of seven former soccer and three of four former rugby players’ brains, often in combination with other neurodegenerative pathologies, including AD, DLB, cerebrovascular disease, and ARTAG. The authors concluded that CTE occurs in a high proportion of former soccer and rugby players dying with dementia, although their novel system of integrated diagnosis often considered the dementia related to non-CTE pathologies [63]. CTE pathology was also reported in five of nine middle-aged rugby union players in the Australian Sports Brain Bank [16, 115]. Microscopic examination revealed pathognomonic lesions of CTE in the bilateral frontal, temporal, and parietal cortices.

### Australian rules football

Six of eight Australian Rules football players whose brains were donated to the Australian Sports Brain Bank were diagnosed with CTE by Suter et al. [115].

### Ice hockey

Schwab et al. analyzed the brains of 24 football and 11 ice hockey players [105]. Neuropathological evaluation found that 17/35 football and hockey players were diagnosed with CTE, including 6 of the 11 ice hockey players. Other comorbid neuropathologies were present in 13 athletes.

### CTE in military personnel

In addition to the reports of Omalu [95] and Goldstein [40], Priemer et al. examined 225 consecutive brains from military service members donated to the Department of Defense-Uniformed Services University Brain Tissue Repository, a brain bank of unselected service members [101]. The brain donors included eight women (3.5%), were relatively young (male donors’ average age: 48.2 years, range 18–87; female donors’ average age: 47.8 years, range 20–63), and included active duty and retired personnel. The study was limited by a lack of standardized clinical assessment of TBI or RHI history and non-standardized neuropathological evaluation. The number of tissue blocks sampled varied from 5 to 23 samples (average 13), and guidelines recommended by the NINDS–NIBIB consensus panel for assessment of CTE were not routinely followed [74]. CTE was found in 10 of 225 donors (4.4%), average age 54.8 years, and range 31–87 years. Although the authors considered CTE in this population rare, the prevalence of 4.4% is many times higher than that found in community brain banks [73, 114], despite the young age and broad range of TBI or RHI exposures in the sample. All ten CTE cases had a history of contact sport play. Of the 45 individuals with history of blast exposure, 3 were diagnosed with CTE (6.7%). Of the 21 with history of military-related TBI, 3 were diagnosed with CTE (14.3%) and 8 of the 44 with history of civilian non-sports-related TBI were diagnosed with CTE (18.1%). None of those with non-contact sports exposure were found to have CTE. The authors did not specify how many donors saw combat in the military.

### Community-based studies and brain bank series

In older community-based populations, the diagnosis of CTE is rare. Adams et al. studied 164 brain donors from the Framingham Heart Study (FHS) Brain Bank, a community-based aging cohort [3]. FHS brain donors were included in the study if their next of kin completed an extensive standardized RHI and sports questionnaire. Of the 164 FHS brain donors who qualified (mean age at death: 87.2 ± 0.8 years), one (0.6%) was diagnosed with CTE, a man who played 8 years of high school and college football. Another study of an older European community-based population, the Vienna Trans-Danube Ageing study, evaluated 310 brain donors ranging in age from 76 to 91 years, mean 83 ± 6.3 years, comprising 181 females and 129 males, and found no case that fulfilled diagnostic criteria for CTE (0%) [34]. In contrast, ARTAG was identified in 117 cases (38%), including isolated ARTAG pathologies at the depths of cortical sulci in 25 cases (8%). In a large retrospective cohort study of 2566 autopsy cases in the Mayo Clinic Tissue Registry, online obituary and high school yearbook records were queried for participation in contact sports resulting in identification of 300 former athletes and 450 non-athletes [10]. In these 750 cases, neocortical tissue was screened for CTE pathology, blinded to exposure, or demographic information. Cases with pathology that fulfilled consensus criteria for CTE were classified “CTE positive”. “Features of CTE” was used to designate cases with pathology suggestive of CTE without fitting all parts of the criteria, and exclusive of AD and ARTAG. Together, features of CTE or CTE-positive pathology were observed in 5.6% of the cohort; if limited to only CTE positive, CTE pathology was found in 2.8% of subjects, all of whom were male. Of those with CTE pathology, 15 were former contact sport athletes (71.4%) and 6 were not (28.6%). In 532 consecutive brain autopsies from the community-based Adult Changes in Thought (ACT) study, a longitudinal population-based prospective cohort study of brain aging and incident dementia, mean age at death 86.9 ± 6.9 years, three cases (0.6%) were found to have CTE [100]. All three cases had low-stage CTE pathology, no information regarding contact sport history or neurotrauma during military service was available, although none had a history of TBI with loss of consciousness. In an autopsy series of 180 consecutive brain referrals to the Royal Prince Alfred Hospital in Sydney, Australia, including 18 cases donated with a clinical diagnosis of dementia, p-tau immunohistochemistry on cortical tissue blocks revealed 4 cases of low-stage CTE, 3 males and 1 female (2.2%). One was a former boxer, and another had a history of TBI with complications; two cases had a long history of treatment-resistant tonic clonic epilepsy. McCann et al. screened 636 cases from the Sydney Brain Bank, a collection that includes neurodegenerative disease and neurologically normal controls, for CTE [73]. Five cases (0.8%) showed CTE, three of whom had a history of TBI and high exposure to RHI from contact sports. Two cases had no known history of TBI or RHI in sports, although information regarding previous contact sport participation was not systematically collected.

### Other exposures

CTE has also been reported as a comorbidity in specific autopsy-confirmed disorders, including older individuals in the setting of neurodegenerative diseases associated with frequent falls, such as multiple system atrophy (6%) [56] and PSP [67], and in contact sport athletes [77] and veterans diagnosed with ALS, many of whom experienced neurotrauma (6%) [119]. CTE has also been diagnosed in a young woman with a history of interpersonal violence [29], and in a young man with head-banging behaviors since childhood [64]. CTE has been reported in poorly controlled grand mal seizures [51]. Of ten patients who underwent epilepsy surgery, median age at resection 32.5 years, pathological examination showed focal sparse tau-immunoreactive lesions along the sulcal depths in the resected frontal lobe of one patient (10%). In a subsequent study of 60 consecutive patients who had undergone surgical treatment for drug-resistant focal epilepsy between 18 and 45 years of age, median age at resection 29.5 years, none of the patients had pathological findings characteristic of CTE (0%), although 23 patients (38%) demonstrated non-specific p-tau pathology, including neurites, pretangles, NFTs, subpial tau, and glial tau [109].

### CTE in the absence of a history of RHI or other neurotrauma

The rarity of CTE in community-based populations and brain banks using NINDS–BIBIB criteria (0.0%, 0.6%, 0.6%, 0.8%, 2.2%, 2.8%) [3, 10, 34, 73, 100, 114] stands in contrast with a study by Iverson et al., who evaluated a postmortem series of eight older men, age range 56–82 years, mean 71 years, who were autopsied as part of the Tampere Sudden Death Study in Tampere, Finland [49]. RHI history was determined by a retrospective survey; however, the survey did not ask directly if the decedent played contact sports, including soccer, the most played sport in Finland. Neuropathological examination concluded that six of the eight cases (75%) had “sparse” “pathognomonic lesions” of CTE. Subsequent review of the CTE pathology provided in the figures of the manuscript by five neuropathologists experienced with CTE and ARTAG considered the p-tau pathology to represent ARTAG or non-specific changes, and non-diagnostic for CTE [78]. Iverson et al. conclusion that “CTE pathology is present in people not known to have experienced repetitive neurotrauma,” therefore, is in question. The individuals were not known to have experienced neurotrauma in part due to incomplete contact sport histories and the pathology did not convincingly demonstrate diagnostic CTE lesions.

Several other studies have reported CTE in individuals with no known history of neurotrauma, although the case numbers are small, some studies did not include adequate data on RHI history, and some might have misinterpreted non-specific astrocytic accumulations of p-tau or ARTAG as diagnostic for CTE [35, 107, 118]. Shively et al. reported the neuropathological findings in five institutionalized patients with schizophrenia and a history of surgical leucotomy with at least 40 years of survival. In the descriptions and images provided, there appears to be subpial ARTAG at the depths of the sulci, but there is no evidence of a diagnostic pathognomonic CTE lesion. In addition, little is known about the patients’ clinical status, such as whether the leukotomy predisposed them to frequent falls or poorly controlled seizures, conditions associated with RHI. Tribett et al. reported the neuropathological findings in a 63-year-old man who had suffered a gunshot wound to the head in his early 20s, causing hemiplegia and post-traumatic epilepsy [118]. No details were provided on how well his epilepsy was controlled. From the description and tau immunohistochemistry illustrated, the density of the p-tau pathology is increased at the depths of a sulcus, but does not appear to include a pathognomonic lesion. Gao et al. reported a 45-year-old man with CTE-like neuropathology and ALS without a known antecedent history of trauma [35]. Tau immunohistochemistry showed NFTs and neuropil threads in the frontal and temporal cortices with a predilection for the superficial cortical layers and depths of the sulci, as well as astrocytic p-tau pathology; however, the authors do not describe a pathognomonic lesion. Additionally, NFTs were described in the hippocampus and entorhinal cortex, yet no p-tau pathology was found in the amygdala, nucleus basalis of Meynert, or LC, an unusual distribution for CTE.

## Molecular, biochemical, and cellular aspects of p-tau pathology in CTE

**Fig. 4:**
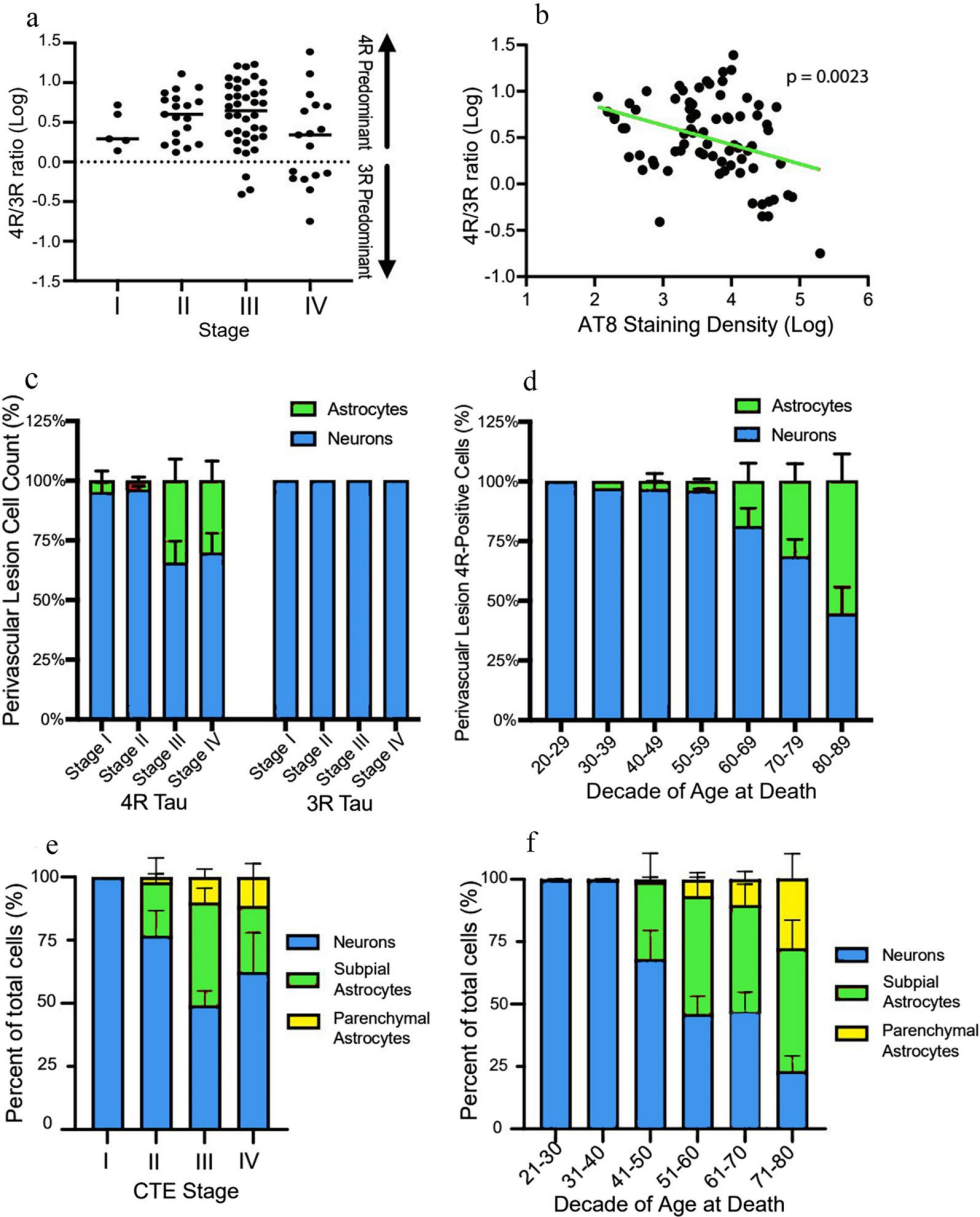
The evolution of 3R/4R tau and astrocytic involvement in CTE pathology (adapted with permission from [21] and [17]). **a** Quantitative measurement of the relative ratios of 4R/3R p-tau present in the dorsolateral frontal cortex at the depth of the cortical sulcus across the stages of CTE. Values over 0 contain more 4R tau while values under 0 contain more 3R tau. Although 4R predominates at all stages of disease, there is relatively more 3R tau with increasing CTE stage. In cells containing 4R tau in CTE stage I and II, 95.8% ± 10.2% and 96.1% ± 4.7% were neurons, respectively. For CTE stage III and IV, 65.6% ± 28.7% and 69.7% ± 26.0% of cells containing 4R were neurons, respectively [21]. **b** Linear regression analysis of the 4R/3R ratio compared to the AT8 p-tau staining density in the dorsolateral frontal cortex. Data is log transformed. Each dot represents one case. As AT8 pathology accumulates, there is a decreasing ratio of 4R/3R p-tau [21]. **c** Quantitative measurement of the relative percentages of neurons and astrocytes that contain p-tau in the pathognomonic CTE lesion. Neurons contain both 3R and 4R while astrocytes only contain 4R ptau. Neurons were the primary cell type associated with the CTE lesion. Further ANCOVA analysis demonstrated that CTE stage was not significantly correlated with the percentage of 4R astrocytes around the lesion when including age at death as a covariate in the model (*p* = 0.031), suggesting age is a stronger driver of p-tau astrocytes than CTE stage. **d** Relative percentages of neurons and astrocytes in pathognomonic lesion that contain 4R tau stratified by decade of age at death. Data is presented as mean ± SD. There is a significant increase in astrocytic 4R p-tau with age at death, most prominent over age 60. **e, f** Percentage of neuronal, subpial astrocytic, and parenchymal astrocytic p-tau densities separated by CTE stage (**e**), and decade of age (**f**) further demonstrating the presence of p-tau positive astrocytes in the parenchyma are driven more by age than disease stage. Subpial astrocytes are also driven by age, first appearing at age > 40 years. Bar graphs represent mean ± standard error of the mean

Recent neuropathological studies on CTE have focused on the nature of the p-tau pathology, the role of 3R and 4R tau isoforms, and the presence of astrocytes in the CTE lesion. CTE p-tau consists of all six isoforms, 3R and 4R tau, like AD [104]. In 2019, Falcon et al. used cryo-electron microscopy (EM) of p-tau filaments from three subjects with pathologically verified CTE, two boxers and a football player, to show that CTE tau fibrils have a unique molecular configuration consisting of an ordered core of two identical C-shaped protofilaments with a hydrophobic cavity enclosing an unidentified molecule [30, 106]. The cryo-EM structure of CTE tau is distinct from AD, PSP, CBD, and Pick’s disease, and further supports that CTE is a unique tauopathy, ultrastructurally, as well as microscopically. Cherry et al. performed a detailed characterization of tau isoforms in brain donors with a range of CTE stages (*n* = 99) [21]. Immunohistochemical studies revealed both 3R and 4R p-tau accumulation in the pathognomonic lesions, with 4R predominant in CTE stages I–II, and a shift in the 3R/4R ratio toward increased 3R in stages III–IV (Fig. 4). Neurons were found to contain both 3R and 4R, astrocytes contained only 4R, and 4R-positive cells in the pathognomonic lesion in early-stage CTE were exclusively neuronal, with increasing percentages of astrocytes in brain donors ≥ 60 years (Fig. 4). The contribution of immunostaining with different antibodies to the identification of CTE pathology was reported by Ameen-Ali et al. [6]. Adjacent cortical tissue sections from 12 donors with CTE (5 with comorbid neurodegenerative pathology) and AD (*n* = 7) were stained for 3R tau, GT-38, 4R tau, and PHF-1. The stained sections were randomized and independently assessed by a panel of expert neuropathologists, blind to patient clinical history and primary antibody, who recorded whether CTE was present. In sections stained for 4R tau or PHF-1, consensus recognition of CTE was high; in contrast, consensus recognition of CTE in sections stained for 3R tau or GT-38 was poor. The authors concluded that since recognition was poor with 3R and GT-38 immunostaining and that 3R and GT-38 stain only neurons, it is the presence of astroglial tau pathologies that facilitate the detection of CTE pathology. However, an alternative interpretation is that the PHF-1 (which recognizes 3R and 4R tau) and 4R tau (the predominant isoform of neuronal p-tau in CTE) antibodies have greater affinity for CTE p-tau pathology compared to the 3R tau and GT-38 antibodies.

To further investigate the contribution of astrocytes and neurons to CTE pathology, Butler et al. quantitated p-tau pathology in the DLF cortex of 150 individuals with CTE, ages 21–80 years old, without comorbid neurodegenerative pathology [17]. Unlike morphology-based assessment to identify cell types, multiplex immunofluorescent staining was performed to definitively identify p-tau containing cells. P-tau cells were quantitated in the gray matter sulcus, sulcal crest, subpial region, and within pathognomonic CTE lesions. Significantly more neuronal than astrocytic p-tau was found across all cortical regions (*p* < 0.0001). Sulcal astrocytic p-tau was primarily (75%, *p* < 0.0001) localized to subpial regions as TSA, or ARTAG. Neuronal p-tau was significantly associated with age, years of RHI exposure, and CTE severity; astrocytic p-tau pathology was significantly associated only with age (Fig. 4).

Hippocampal p-tau pathology has been shown also to differentiate CTE from AD and PART [20, 31]. Using immunofluorescent analysis and antibodies to antibodies to RD3 (3R Tau) (courtesy of Rohan de Silva), ET3 (4R Tau) (courtesy of Peter Davies), and AT8 (Pierce Endogen), CTE was found to have high levels of p-tau in hippocampal subfields CA2 and CA3 compared to CA1, using all three antibodies. There were also low levels of p-tau in the subiculum compared to CA1 in CTE. In contrast, AD had higher levels of p-tau in CA1 and subiculum compared to CA2/3. Direct comparison of the p-tau burden between AD and CTE demonstrated that CTE had higher p-tau densities in CA4 and CA2/3, while AD had elevated p-tau in the subiculum. Farrell et al. measured p-tau burden using positive-pixel counts on neuroanatomically segmented hippocampal tissue immunostained for AT8 from subjects with CTE and PART [31]. CTE subjects had a higher total p-tau burden compared to PART subjects in all hippocampal sectors. Within groups, PART had significantly higher total p-tau burden in CA1/subiculum compared to CA3 and CA4 and total p-tau burden in CA2 trended higher than CA4. When controlling for p-tau burden across the entire hippocampus, CA3 and CA4 had significantly higher p-tau burden in CTE compared to PART.

## Relationship between RHI, CTE, TDP-43, and ALS

**Fig. 5:**
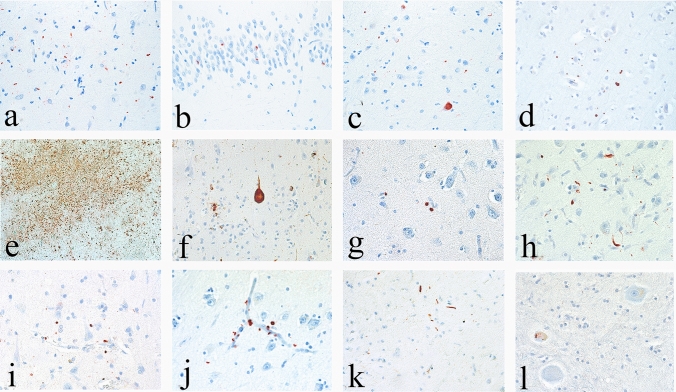
TDP-43 pathology in CTE. Immunoreactivity for phosphorylated TDP-43 is common in CTE and consists of neuronal inclusions and neurites. Representative images of TDP-43 immunoreactive profiles in CTE cases: **a** CA1 hippocampus **b** dentate nucleus of the hippocampus **c** amygdala **d** frontal cortex. All magnifications × 400. TDP-43 immunoreactivity is also found in CTE cases with comorbid ALS**. e–l** A 49-year-old former NFL player with ALS and CTE stage IV. **e** A large CTE lesion at the depth of the dorsolateral frontal sulcus (AT8 immunostain, magnification × 40) **f** Betz cell in motor cortex with NFT (AT8-immunostain, magnification × 200) **g** TDP-43 immunoreactive inclusion and neurites in **g** amygdala, **h** dorsolateral frontal cortex **i** perivascular temporal cortex, **j** perivascular amygdala **k** lumbar spinal cord and **l** dentate nucleus of cerebellum. **g–l** All magnifications × 400

Although CTE is defined by a unique pattern of p-tau pathology, other neurodegenerative pathologies may be affected by RHI exposure and CTE, including beta-amyloid plaques [111], cerebral amyloid angiopathy [110], and Lewy body disease [3]. TDP-43 proteinopathy has been described in a subset of subjects with CTE [79] and includes intraneuronal cytoplasmic inclusions, dot-like and neuritic thread-like inclusions [43, 55, 63, 77] that are occasionally found around blood vessels in the sulcal depths and may co-localize with p-tau deposits [79]. The first NINDS–NIBIBS consensus panel considered TDP-43 immunoreactive neuronal cytoplasmic inclusions and dot-like structures in the hippocampus, anteromedial temporal cortex, and amygdala to be distinctive and a supportive, non-p-tau-related feature of CTE (Fig. 5) [74]. A recent study by Nicks et al. found TDP-43 inclusions in 87 of 307 CTE cases (30.5%) without hippocampal sclerosis (HS), most commonly in the frontal lobes and limbic regions or hippocampus alone and less often in amygdala or frontal regions alone [89]. Hippocampal sclerosis (HS) was reported in 90 of 401 CTE cases (23.4%), and of those, TDP-43 pathology was present in 95.7%. In CTE with HS, TDP-43 inclusions were most common in the frontal lobes and limbic regions or hippocampus alone and, less often, amygdala alone. Participants with CTE and HS were significantly younger (mean 77.7 years) than those with CTE without HS (86.6 years). CTE with HS was associated with greater RHI exposure (reported as contact sport playing years) compared to those with CTE without HS. Although TDP-43 pathology in CTE bears some similarities to limbic-predominant age-related TDP-43 encephalopathy neuropathologic changes (LATE-NC), it was recently recommended that the diagnosis of LATE-NC be avoided in CTE cases until future studies determine whether TDP-43 pathology in CTE is unique to CTE or a manifestation of early-onset LATE-NC [86].

In addition, amyotrophic lateral sclerosis (ALS) with TDP-43 pathology has been reported in contact sports athletes with CTE (Fig. 5) [76, 77, 79, 119]. In a Department of Veterans Affairs ALS brain bank, a tissue repository that collects antemortem disease progression data and postmortem central nervous system tissue from veterans with ALS, neuropathologically confirmed ALS and CTE were reported in 5.8% of 155 brain donors [119]. Individuals with ALS and CTE were more likely to have a history of TBI and to have more severe tau pathology in the frontal cortex and spinal cord. In the ALS plus CTE cases, the most common exposures to RHI included contact sports and military service. Clinically, individuals with ALS and CTE were more likely to have bulbar-onset ALS, behavioral and/or mood changes than those with ALS alone.

ALS risk also has been studied in athletes with high exposure to RHI [28, 65, 88]. Lehman et al. examined mortality in the 3439 athletes who played at least 5 years in the National Football League (NFL) and found that there was an ALS age- and race-standardized mortality ratio of 4.04 (95% CI 1.48–8.79), using US male mortality rates as the standard. Nguyen et al. examined mortality rates in a subset of these same NFL athletes, comparing them with 2708 former Major League Baseball players who played at least five seasons [88]. They reported a race-adjusted ALS hazard ratio of 3.10 (95% CI 0.84–11.38). Daneshvar et al. studied 19,423 male former and current NFL players (age range 23–78 years), followed for a mean of 30.6 years. 38 players were diagnosed with ALS, and 28 died during the study period, representing a significantly higher incidence of ALS diagnosis (standardized incidence ratio 3.59; 95% CI 2.58–4.93) and mortality (standardized mortality ratio 3.94; 95% CI 2.62–5.69) among NFL players compared with the US male population, adjusting for age and race [28]. Moreover, athletes with ALS had longer NFL careers than those without ALS, suggesting an association between NFL duration of play or RHI exposure and ALS.

## The clinical syndrome associated with CTE pathology or traumatic encephalopathy syndrome (TES)

Advances in understanding the clinical syndrome associated with CTE pathology have been made primarily through retrospective interviews with informants of individuals diagnosed at autopsy with CTE, as well as in vivo studies with participants who are at high risk for CTE, such as former NFL players. The clinical features associated with CTE pathology include cognitive, mood, behavior, and motor impairments, with dementia common in severe disease. Younger individuals tend to present with mood and behavior symptoms, whereas older individuals more commonly present with cognitive impairment and executive dysfunction [85, 112]. The mood and behavioral symptoms can be difficult to distinguish from depression, anxiety, mental health disorders, and post-concussion syndrome, and the cognitive decline bears similarities to that of Alzheimer's disease. In 2014, based on literature review of symptoms in neuropathologically validated CTE cases, research diagnostic criteria for traumatic encephalopathy syndrome (TES) were proposed to diagnose CTE pathology in life [85]. The validity of the 2014 TES criteria was assessed in 336 consecutive brain donors by Mez et al. and was found to have good sensitivity (0.97), but low specificity (0.21) [80]. Cognitive symptoms were significantly associated with CTE pathology, but mood/behavior or motor symptoms were not [80]. Requiring the presence of cognitive symptoms decreased sensitivity somewhat (0.90) with substantial improvements in specificity (0.48). In 2019, the First NINDS Consensus Workshop to Define the Diagnostic Criteria for TES, a multidisciplinary panel of 20 clinician–scientists and 7 observers, was convened to develop the NINDS Consensus Diagnostic Criteria for TES using the 2014 criteria as a starting point [52]. After four rounds of reviewing, anonymous voting, and revising, a consensus was reached for four primary criteria for the diagnosis of TES as well as criteria for supportive features and provisional levels of certainty for CTE pathology. The four primary criteria for the NINDS Consensus Diagnostic Criteria for TES, published in 2021, include: (1) substantial exposure to RHI; (2) core clinical features of cognitive impairment (episodic memory and/or executive functioning) or neurobehavioral dysregulation (explosiveness, impulsivity, rage, violent outbursts, and emotional lability), or both, and a progressive course; (3) clinical features not fully accounted for by other disorders. For those meeting the first three criteria for TES, 4) a level of functional dependence and dementia is graded according to descriptions of the following levels: independent, subtle/mild functional limitation, mild dementia, moderate dementia, or severe dementia. The diagnosis of TES, a clinical syndrome, is intended for clinical research settings, and is not designed to represent a diagnosis of CTE, a neuropathologic diagnosis [52]. It is anticipated that the accuracy of the diagnosis of TES will be considerably improved as fluid and neuroimaging biomarkers of CTE pathology are developed.

Although it remains unclear whether all the core clinical features of TES are correlated with CTE p-tau pathology, especially in early stage CTE, multiple studies have shown that antemortem dementia is consistently correlated with quantitative and semi-quantitative p-tau pathology in CTE and CTE stage [4, 5, 22]. Alosco et al. analyzed that 359 brain donors with CTE and found that 216 (60.2%) had dementia [4]. The presence of dementia followed an age-dependent progression, and older age at death was associated with increased odds for having dementia. For every level increase in CTE stage, there was a 1.64X increased odds for antemortem dementia at the time of death. The statistically significant association between CTE stage and dementia remained after controlling for comorbid neurodegenerative disease, arteriolosclerosis, white matter rarefaction, education level, racial identity, and age at death.

## Relationship between RHI and CTE

Over recent years, the question of causation in CTE has become a topic of fierce debate, given the rise in reports of CTE in former American football, ice hockey, rugby, and soccer players, and the financial implications of multiple lawsuits brought by former players against sports organizations. Uncertainty regarding a causal relationship between RHI and CTE is largely founded on concerns for selection bias in brain bank populations, limitations inherent to cross-sectional neuropathological studies and case series, and ambiguity regarding the clinical spectrum of TES, a clinical diagnosis defined primarily through retrospective interviews with informants of individuals diagnosed postmortem with CTE.

The ideal research design to ascertain a causal link between RHI and CTE would be a large-scale, prospective, longitudinal study of well-characterized individuals exposed and unexposed to American football followed to death with postmortem neuropathological analysis, a study that would require six or seven decades of observation, and would be unethical, in view of the probability of harm to at least some of the participants [18, 33]. Nowinski et al. recently applied the Bradford-Hill criteria to the scientific literature on CTE to explore the line of reasoning for a causal relationship between RHI and CTE. [91] The Bradford-Hill criteria are the most frequently cited framework for causal inference in epidemiologic studies and include nine aspects of association by which to gauge epidemiological evidence for causation: strength of association, consistency, specificity, temporality, biological gradient, plausibility, coherence, experiment, and analogy [32]. Nowinski et al. advocated for strength of association by extrapolating from multiple brain bank studies and showing highly significant ORs, ranging from 2.6 to 901.9, for CTE in brain donors with RHI exposure compared to non-RHI controls, with the wide range in ORs likely accounted for by varying levels of RHI exposure and cohort composition across the studies [3, 10, 12, 79, 83, 101]. Consistency has been demonstrated across multiple independent, international studies that found an association between RHI exposure of many types and CTE pathology [1, 3, 4, 7, 10, 12, 16, 29, 42–44, 51, 53, 56, 63, 64, 67, 68, 73, 75, 79, 89, 92, 94, 96–98, 101, 105, 113–115, 119, 120]. Specificity is supported by the fact that RHI exposure is the only known unifying factor (present in 97%) among the over 600 CTE cases reported in the literature to date (Table 4). Temporality is supported by RHI exposure occurring before, often decades before, the onset of dementia in subjects who were later found at autopsy to have CTE. Coherence is reinforced by the lack of any other conflicting evidence to support the development of CTE pathology. Plausibility requires that the association between RHI and CTE pathology can be explained by biological models such as computational, finite element and animal models of head impact injury, of which there is compelling recent evidence. The Centers for Disease Control and the NINDS have formally updated their resources to reflect the understanding that CTE is caused in part by repeated brain injuries [19, 90].

### Models of head impact injury

Computational models of head impact injury have shown that the strain, strain rate and mechanical deformation of the brain is greatest at the depths of the cortical sulci and around small blood vessels, the hallmark regions of p-tau deposition in CTE [38, 121]. A mesoscale finite element analysis that simulated an American football player wearing a helmet also found peak stresses in the cortical sulci [9]. The sulcal geometry produced complex stress waves that interacted with each another to create increased shear stresses at the sulcal depths that were significantly larger than in areas without sulci. Recently, Braun et al. used computational modeling to show that the mechanical energy associated with high-strain rate deformation alone can induce tau mislocalization to dendritic spines and synaptic deficits in cultured rat hippocampal neurons [14, 15]. Kerwin et al. demonstrated mechanical strain at the depths of the sulci from fluid cavitation expansion during repetitive low-force impacts on computational models, adding further evidence that mechanical interactions during impact injury could contribute to the location of tau pathology in CTE [54]. The perivascular deposition of p-tau might be explained by blood–brain barrier injury, which has been shown to be related to high strain [108], or the effects of displacement across a field with tissue inhomogeneity created by the presence of a blood vessel [23]. In addition, two-dimensional quasi-static, finite element models of a perivascular region show strain is maximum nearest the vessel during brain deformation [14].

### Animal models

While studies of rodent brains exposed to blast or repetitive concussive impacts have shown increased p-tau deposition [40, 57, 116], rodent models have not produced focal perivascular p-tau aggregates, i.e., the pathognomonic CTE lesion. There are many likely explanations for this, including that rodent brains are not gyrencephalic, thus, lack the sulcal anatomy that is susceptible to sheer stress, and that mouse and human tau have only 89% amino acid homology, 3R tau is absent in adult mice, and the n-terminal sequence of human tau contains a tau peptide (residues 17–28) that the mouse does not. [45] Recently, Ackermans et al. analyzed postmortem brains of three muskoxen and four bighorn sheep for evidence of neurodegeneration [2]. Musk oxen and big horn sheep have gyrencephalic brains and participate in combative headbutting. In the musk oxen, high densities of p-tau aggregates in neurons, neuritic threads, and neurites were found in the superficial cortical laminae, at the depths of the sulci, and occasionally around blood vessels, appearing very similar to early-stage human CTE.

Yet, the most persuasive recent evidence for a causal relationship between RHI and CTE comes from dose–response studies that support a biological gradient, another criterion of Bradford-Hill. In the 2013 McKee study, of 35 brain donors who played football and were diagnosed with CTE, the number of years played (Spearman’s test, *r* = 0.805, *p* < 0.0001), years since retirement (Spearman’s test, *r* = 0.753, *p* < 0.001), and age at death (Spearman’s test, *r* = 0.806, *p* < 0.0001) were significantly correlated with CTE stage, supporting a dose–response between cumulative years of playing football and CTE [79]. This same dose–response was replicated in the 2020 Alosco study [4]. Among 305 brain donors whose primary sport was football, more years of football play was associated with increased odds for having a higher stage of CTE (OR 1.10, 95% CI 1.06–1.15, *p* < 0.001), controlling for age at death.

To address the issue of selection bias in the UNITE Brain Bank, Mez et al. evaluated the relationship between of years of football played and CTE status in a convenience sample of 266 deceased American football players from the UNITE and FHS Brain Banks [82]. In total, 223 of 266 participants met neuropathological diagnostic criteria for CTE. More years of football played were associated with having CTE (OR 1.30 per year played, 95% CI 1.19–1.41; *p* = 3.8 × 10^−9^) and with CTE severity (severe vs mild; OR 1.14 per year played, 95% CI 1.07–1.22; *p* = 3.1 × 10^−4^). Moreover, the odds of developing CTE doubled for every 2.6 years of football played. To account for selection bias, the authors used inverse probability weighting implemented as a sensitivity analysis, utilizing simulation approaches to vary the effects of exposure and CTE status. The results showed that at all levels of selection bias, the strength of the duration of play-CTE relationship remained consistent. To further account for selection bias in the UNITE Brain Bank, LeClair et al. extended existing epidemiological methods applicable to brain donation studies susceptible to selection bias and evaluated the relationship between level of American football playing and CTE diagnosis [62]. The LeClair study sample included 290 deceased male former American football players who donated their brains to the UNITE Brain Bank between 2008 and 2019. After adjustment for selection bias, college-level and professional football players had 2.38 [95% simulation interval (SI) 1.16–5.94] and 2.47 (95% SI 1.46–4.79) times the risk of being diagnosed with CTE as high school-level players, respectively; these estimates are larger than estimates with no selection bias adjustment. These findings suggest that the selection bias in the UNITE study might be causing an underestimation of the risk of CTE. This study also provides evidence to support a dose–response relationship between American football playing and CTE and ensures a generalizable estimate of the association between RHI and CTE.

In contrast, Schwab et al. analyzed postmortem brains of 35 male athletes, including 24 (68.6%) former football players and 11 (31.4%) former hockey players for the presence of CTE pathology [105]. In total, 17 of 35 former players (48.6%) showed pathologic evidence of CTE. Among the hockey players, CTE was diagnosed in 4/7 (57.1%) forwards and 2/4 (50%) defensemen. There was no correlation between position played, hockey fighting/penalization histories, career duration, or retirement age and CTE. The methods, however, were limited by low statistical power, inaccurate assessments of career duration (measured only as age at retirement), and the combination of football players with ice hockey players, sports with very different RHI exposure profiles [81, 93] A recent study demonstrated a dose–response relationship between duration of ice hockey play and CTE risk and severity in a sample of 74 consecutive brain donors to the UNITE and Framingham Heart Study brain banks [1]. Each additional year of ice hockey played corresponded to a 23% increase in odds of being diagnosed with CTE and a 15% increase in odds of increasing one stage of CTE severity (0–IV). When controlling for hockey as the primary sport of exposure (*n* = 56), the results were similar, indicating that increased duration of ice hockey play might increase risk for CTE.

## Summary

CTE is a distinctive pathology characterized by neuronal p-tau aggregates that are focal, perivascular, and cortical in mild disease in young individuals and widespread and diffuse in severe disease and in older individuals. CTE tau consists of a distinctive molecular structural configuration of p-tau fibrils that is unlike the changes observed with aging, Alzheimer's disease, or any other tauopathy. Since the publication of NINDS–NIBIB criteria for the neuropathological diagnosis of CTE in 2016 and refined criteria in 2021, multiple studies conducted by independent, international groups investigating different populations have found CTE pathology in individuals with a history of RHI from various sources. Conversely, studies involving large community populations and brain banks have found CTE pathology to be rare or absent (0–2.8%). Multiple experimental models have demonstrated that tissue strain and physical deformation is greatest in the perivascular and sulcal depth regions after head impact injury, accurately predicting the location of p-tau deposits. In addition, there is a robust dose–response relationship between CTE and years of American football play. The preponderance of the evidence suggests a high likelihood of a causal relationship between RHI and CTE, a conclusion that is strengthened by the absence of any evidence for plausible alternative hypotheses. There is no other common variable, aside than RHI, that explains why so many contact sports players worldwide, playing diverse sports, have been diagnosed with CTE, while individuals without RHI exposure have not [91]. Nevertheless, there remain areas of uncertainty in CTE and topics that require future investigation. These include better distinction of mild CTE pathology from the changes of ARTAG and better detection of CTE pathology in the presence of a co-existing tauopathy such as AD. In addition, although dementia correlates with severity of p-tau pathology in CTE and CTE stage, the clinical symptoms associated with CTE pathology, or TES, especially the mood and behavioral symptoms, need better specificity, especially in mild stages of disease. To improve detection and diagnosis of the clinical syndrome of TES, prospective, longitudinal studies of individuals at risk for CTE are needed, along with robust fluid and imaging biomarker studies, and analysis of other pathologies known to occur in CTE, including neuroinflammation, small vessel changes, and white matter degeneration.

## Footnotes

1 CTE is sometimes referred to as CTE-Neuropathologic Change (CTE-NC). However, CTE as currently defined refers to a pathology, whereas TES is the clinical syndrome associated with CTE pathology.
